# Evolving clinical features of *Mycoplasma pneumoniae* infections following COVID-19 pandemic restrictions: a retrospective, comparative cohort study

**DOI:** 10.1007/s00431-025-06326-y

**Published:** 2025-08-07

**Authors:** Elena Robinson, Michael Zellner, Ester Osuna, Michelle Seiler, Martin Theiler, Semjon Sidorov, Stefanie von Felten, Christoph Berger, Patrick M. Meyer Sauteur

**Affiliations:** 1 Division of Infectious Diseases and Hospital Epidemiology, Children’s Research Center, University Children’s Hospital Zurich, University of Zurich, Lenggstrasse 30, CH-8008 Zurich, Switzerland; 2 Division of Diagnostic Imaging, University Children’s Hospital Zurich, Zurich, Switzerland; 3 Emergency Department, University Children’s Hospital Zurich, Zurich, Switzerland; 4 Pediatric Skin Center, Department of Dermatology, University Children’s Hospital Zurich, Zurich, Switzerland; 5 Department of Biostatistics at Epidemiology, Biostatistics and Prevention Institute (EBPI), University of Zurich, Zurich, Switzerland

**Keywords:** Community-acquired pneumonia, Epidemiology, Extrapulmonary manifestations, Non-pharmaceutical interventions (NPIs), Severe acute respiratory syndrome coronavirus 2 (SARS-CoV-2)

## Abstract

Since its delayed re-emergence after non-pharmaceutical interventions (NPIs) against the COVID-19 pandemic, *Mycoplasma pneumoniae* has caused community-acquired pneumonia outbreaks worldwide. In this study, we aimed to investigate how the clinical characteristics and severity of *M. pneumoniae* infections have changed after COVID-19 pandemic restriction, in order to enable adequate interpretation of clinical features and response to future *M. pneumoniae* epidemics. This retrospective, comparative cohort study compared clinical features and severity of children with *M. pneumoniae* detection by PCR during the periods April 1, 2015, to March 31, 2020 (pre-NPI); April 1, 2020, to March 31, 2022 (NPI); and April 1, 2022, to March 31, 2025 (post-NPI). Clinical features were compared between periods by Kruskal–Wallis rank sum test or Fisher’s exact test, as appropriate. Moreover, we compared hospitalization and intensive care unit (ICU) admission using generalized linear models. In total, 321 patients were included in the study. Since the first detection of *M. pneumoniae* after the COVID-19 pandemic in summer 2023, the re-emergence has shown a bimodal curve with two distinct peaks (post-NPI first-year and second-year). The median age of patients was higher in the post-NPI than the pre-NPI period (9.05 vs 8.20 years), particularly during the first-year peak (11.00 years). Obstructive diseases were observed more frequently post-NPI compared to pre-NPI (18.6% vs 9.6%). Moreover, more patients presented with chest pain (8.9% vs 2.4%) and pleural effusions (45.7% vs 28.9%) post-NPI than pre-NPI. Conversely, extrapulmonary manifestations were less frequent post-NPI (18.6% vs 30.1%), particularly dermatological (15.7% vs 25.3%) and neurological (1.3% vs 4.8%) manifestations. Hospitalization rate (38.6% post-NPI vs 43.9% pre-NPI) and length of stay (median, 4 [IQR, 2–5] vs 4 [IQR, 3–6] days) were similar, while generalized linear models showed a trend toward fewer hospitalizations post-NPI (odds ratio [OR], 0.72 [95% CI, 0.42–1.23]; *P* = 0.22). The same applied to ICU admission rate (5.1% post-NPI vs 4.9% pre-NPI), with a trend toward fewer ICU admissions post-NPI (OR, 0.90 [95% CI, 0.29–3.34]; *P* = 0.86).

*Conclusion*: We observed notable changes in the clinical presentation of re-emerging *M. pneumoniae* infections compared to the pre-COVID-19 pandemic period, particularly an increase in obstructive phenotypes and pleural effusions. However, overall disease severity appeared to remain largely unchanged.

**Table:** 

**What is Known:** *• The delayed re-emergence of M. pneumoniae in late 2023 was substantial in terms of case numbers across many geographical locations.* *• No statistically increased proportion of severe or worse outcomes of re-emerging M. pneumoniae infections could be observed globally compared with pre-COVID-19 pandemic epidemics.*	
**What is New:** *• Clinical features of M. pneumoniae infections in children partly changed following COVID-19 pandemic restrictions, with new signs like obstructive phenotypes and pleural effusions.* *• The findings suggest that there has been no overall increase in disease severity; in fact, extrapulmonary manifestations were fewer, with trends toward reduced hospitalizations and ICU admissions.*

**Supplementary Information:**

The online version contains supplementary material available at 10.1007/s00431-025-06326-y.

## Introduction

*Mycoplasma pneumoniae* is a common cause of respiratory tract infections in children. The clinical significance of this pathogen has been impressively demonstrated by its re-emergence after COVID-19 pandemic restrictions [1], resulting in community-acquired pneumonia (CAP) outbreaks worldwide [2, 3]. We have previously reported clinical features associated with *M. pneumoniae* infection in children before the COVID-19 pandemic using improved diagnostic methods [4]. In this cohort of children, *M. pneumoniae* infection was confirmed by the detection of pathogen-specific IgM antibody-secreting cells (ASCs) by enzyme-linked immunospot (ELISpot) assay, which distinguishes between infection and carriage [4]. Here, this unique cohort was used as a reference to compare clinical features and the severity of re-emerging *M. pneumoniae* infections.

## Methods

### Study design and population

We conducted a retrospective, comparative cohort study at the University Children’s Hospital Zurich, the largest, tertiary pediatric hospital in Switzerland. Patients from 0 to < 18 years that presented with acute symptoms consistent with *M. pneumoniae* infection and simultaneous detection of *M. pneumoniae* by polymerase chain reaction (PCR) from April 1, 2015, to March 31, 2025, were included.

Patients were assigned based on the PCR test date to the different time periods, defined by the presence of non-pharmaceutical interventions (NPIs) against COVID-19 in Switzerland, as previously described [1]: pre-NPI period, April 1, 2015–March 31, 2020; NPI period, April 1, 2020–March 31, 2022; and post-NPI period, April 1, 2022–March 31, 2025.

Patients were identified by review of electronic medical and laboratory records (Fig. S1) and screened for eligibility. Additionally, patients of the previous cohort (myCAP study [4]) were assessed for eligibility for this study. Detection numbers may differ from publications of the ESGMAC MAPS study [1, 5] as the detailed description of clinical characteristics in this study was subject to general consent.

Demographic, clinical, radiographic, laboratory, and microbiological data were gathered from medical records of included patients and collected and managed using REDCap (REDCap Consortium).

### Statistical analysis

This study follows the STROBE guidelines [6]. We report median and interquartile range (IQR) for continuous variables, frequency and percentage for categorical variables, and missing values. We performed Kruskal–Wallis rank sum tests for continuous variables and Fisher’s exact test for categorical variables, reporting the unadjusted *P* values, to assess differences between the groups. Additionally, we compared the binary outcomes hospitalization and intensive care unit (ICU) admission using binomial generalized linear models with logit link and cohort as explanatory variable. To adjust for potential confounding, we added age, sex, and underlying diseases as explanatory variables.

Analyses were performed with R software, version 4.5.0 (R Foundation for Statistical Computing).

### Ethical considerations

This study was approved by the ethics committee of the Canton of Zurich, Switzerland (BASEC no. 2025–00039). All included patients had provided general consent at the University Children's Hospital Zurich or had given written informed consent for further use of health-related data as part of the previous myCAP study [4] (BASEC no. 2016–00148; Fig. S1).

## Results

**Fig. 1:**
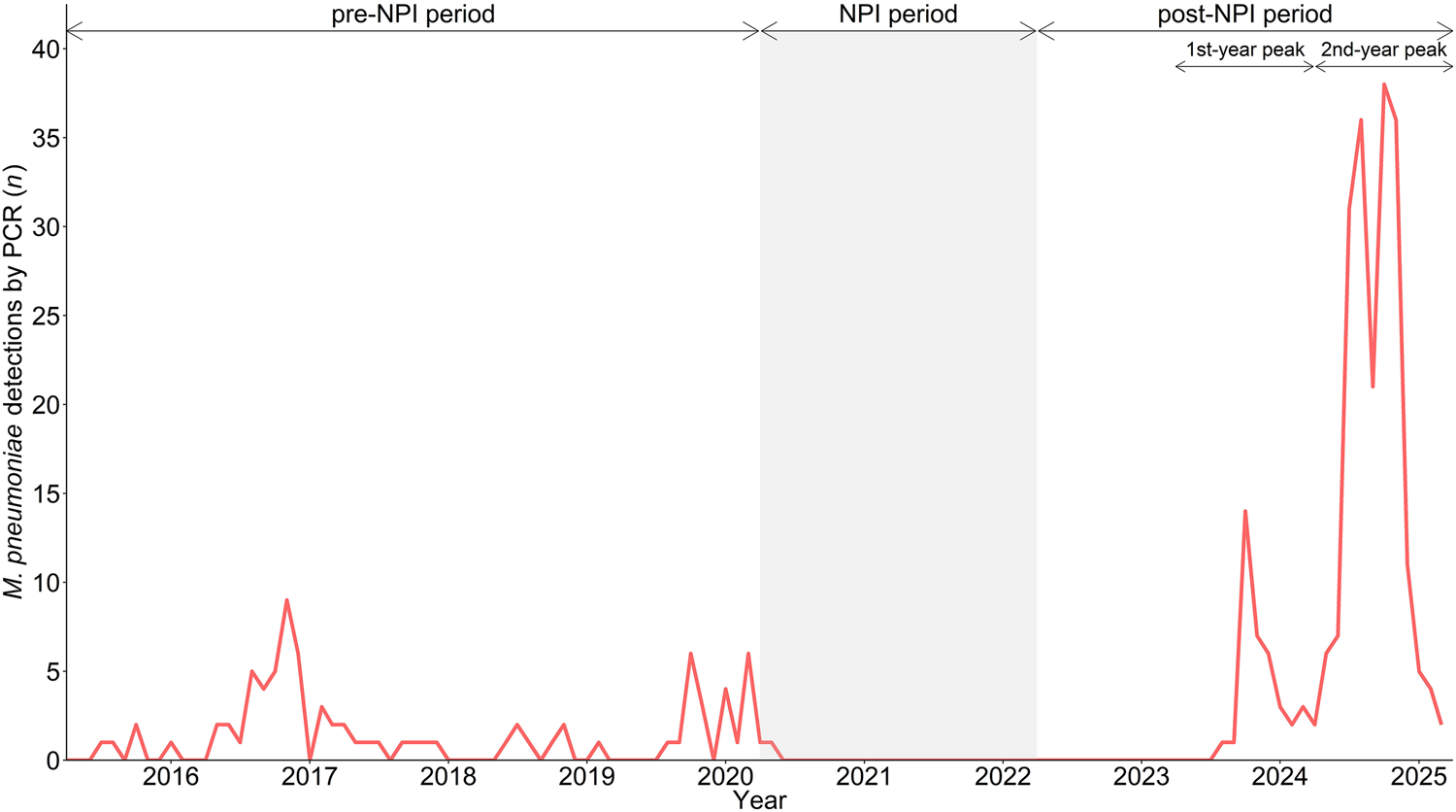
*Mycoplasma pneumoniae* detections by PCR from April 1, 2015*,* to March 31, 2025*,* at the University Children’s Hospital Zurich, Switzerland*.* Detection numbers may differ from publications of the ESGMAC MAPS study [1, 5] as the detailed description of clinical characteristics in this study was subject to general consent. Abbreviations: *NPI* non-pharmaceutical intervention, *PCR* polymerase chain reaction

During the 10-year study period, 321 patients were included, 83 during the pre-NPI period (2015–2020), two during the NPI period (2020–2022), and 236 during the post-NPI period (2022–2025; Fig. S1). Since the first detection of *M. pneumoniae* after the COVID-19 pandemic in summer 2023, the re-emergence has shown a bimodal epidemic curve, with two distinct peaks in post-NPI case numbers (post-NPI first-year peak, 2023–2024, *n* = 37, and post-NPI second-year peak, 2024–2025, *n* = 199; Fig. 1).

Demographic and clinical characteristics are shown in Table 1 and Tables S1–S9. The median age of patients was higher in the post-NPI than the pre-NPI period (9.05 vs. 8.20 years), particularly during the post-NPI first-year peak (11.00 years). Underlying diseases were more common post-NPI than pre-NPI (26.7% vs 13.3%; Table S1). The duration of prodromal symptoms decreased post-NPI compared to pre-NPI (median 7.0 vs 9.0 days; Table 1). The main symptom at presentation was cough (94.0% post-NPI vs 89.2% pre-NPI), and changes in symptom frequency were an increase in chest pain (8.9% vs 2.4%) and diarrhea (8.5% vs 2.4%), and a decrease in fever (28.2% vs 50.0%) and sore throat (8.9% vs. 19.3%).

**Table 1:** Demographic and clinical characteristics of children with *Mycoplasma pneumoniae* detection by PCR from April 1, 2015, to March 31, 2025

Variable	2015–2020 (pre-NPI) (*n* = 83)	2022–2025 (post-NPI) (*n* = 236)	*P* value	2023–2024 (post-NPI first-year peak) (*n* = 37)	2024–2025 (post-NPI second-year peak) (*n* = 199)	*P* value
**Demographic characteristics**						
Age (years)	**8.20 (5.30, 10.20)**	**9.05 (5.45, 11.50)**	**0.30**	11.00 (7.10, 13.50)	8.80 (5.30, 11.00)	0.0019
Sex, female	**33 (39.8%)**	**105 (44.5%)**	**0.52**	18 (48.6%)	87 (43.7%)	0.59
Underlying diseases	**11 (13.3%)**	**63 (26.7%)**	**0.015**	11 (29.7%)	52 (26.1%)	0.69
Family members with RTI^a^	**47 (71.2%)**	**107 (74.8%)**	**0.61**	10 (71.4%)	97 (75.2%)	0.75
NA	**17**	**93**		23	70	
**Clinical characteristics**						
***Symptoms at presentation***						
Rhinitis	**22 (26.5%)**	**66 (28.1%)**	**0.89**	12 (32.4%)	54 (27.3%)	0.55
Sore throat	**16 (19.3%)**	**21 (8.9%)**	**0.016**	7 (18.9%)	14 (7.1%)	0.03
Cough	**74 (89.2%)**	**221 (94.0%)**	**0.15**	36 (97.3%)	185 (93.4%)	0.70
Wheezing	**2 (2.4%)**	**3 (1.3%)**	**0.61**	0 (0.0%)	3 (1.5%)	1.00
Chest pain	**2 (2.4%)**	**21 (8.9%)**	**0.05**	7 (18.9%)	14 (7.1%)	0.03
Nausea and/or vomiting	**16 (19.3%)**	**59 (25.1%)**	**0.37**	14 (37.8%)	45 (22.7%)	0.063
Diarrhea	**2 (2.4%)**	**20 (8.5%)**	**0.077**	6 (16.2%)	14 (7.1%)	0.10
Prodromal symptom duration (days)	**9.0 (6.0, 11.0)**	**7.0 (5.0, 10.0)**	**0.14**	10.0 (6.0, 14.0)	7.0 (5.0, 10.0)	0.044
NA	**1**	**5**		0	5	
***Signs at presentation***						
Fever	**38 (50.0%)**	**64 (28.2%)**	**0.00072**	7 (19.4%)	57 (29.8%)	0.23
NA	**7**	**9**		1	8	
Oxygen saturation < 93%	**21 (35.6%)**	**64 (28.1%)**	**0.27**	12 (33.3%)	52 (27.1%)	0.43
NA	**24**	**8**		1	7	
Dermatological findings	**18 (21.7%)**	**24 (10.3%)**	**0.014**	6 (16.2%)	18 (9.2%)	0.24
Dermatological prodrome (days)	**1.0 (0.0, 2.0)**	**2.0 (0.0, 5.0)**	**0.67**	5.0 (5.0, 5.0)	1.5 (0.0, 4.5)	0.43
NA	**7**	**15**		5	10	
Neurological findings	**3 (3.6%)**	**8 (3.4%)**	**1.00**	1 (2.7%)	7 (3.6%)	1.00
Neurological prodrome (days)	**2.0 (0.0, 10.0)**	**0.0 (0.0, 1.0)**	**0.20**	0.0 (0.0, 0.0)	0.0 (0.0, 1.0)	0.53
NA	**0**	**1**		0	1	
***Radiographic findings***^**b**^						
*Chest radiograph performed*	**77 (92.8%)**	**141 (59.7%)**	** < 0.0001**	30 (81.1%)	111 (55.8%)	0.0036
Pulmonary infiltrate in chest radiograph	**64 (84.2%)**	**120 (85.7%)**	**0.84**	24 (80.0%)	96 (87.3%)	0.38
Infiltrate type			**0.0041**			0.60
Alveolar (consolidation)	**52 (81.3%)**	**114 (95.0%)**		24 (100.0%)	90 (93.8%)	
Interstitial	**12 (18.8%)**	**6 (5.0%)**		0 (0.0%)	6 (6.3%)	
Pleural effusion	**22 (28.9%)**	**64 (45.7%)**	**0.02**	17 (56.7%)	47 (42.7%)	0.22
NA	**1**	**1**		0	1	
***Diagnosis***						
*URTI (without LRTI)*	**1 (1.2%)**	**34 (14.4%)**	**0.00034**	1 (2.7%)	33 (16.6%)	0.023
*LRTI*						
Total	**81 (97.6%)**	**199 (84.3%)**	**0.00073**	35 (94.6%)	164 (82.4%)	0.082
Pneumonia	**77 (92.8%)**	**172 (72.9%)**	** < 0.0001**	28 (75.7%)	144 (72.4%)	0.84
Other	**2 (2.4%)**	**7 (3.0%)**	**1.00**	1 (2.7%)	6 (3.0%)	1.00
Not specified	**2 (2.4%)**	**20 (8.5%)**	**0.077**	6 (16.2%)	14 (7.0%)	0.099
Obstructive component	**8 (9.6%)**	**44 (18.6%)**	**0.059**	5 (13.5%)	39 (19.6%)	0.49
*Extrapulmonary manifestation*						
Total	**25 (30.1%)**	**44 (18.6%)**	**0.043**	11 (29.7%)	33 (16.6%)	0.068
Dermatological	**21 (25.3%)**	**37 (15.7%)**	**0.068**	8 (21.6%)	29 (14.6%)	0.32
Cutaneous involvement	**18 (85.7%)**	**25 (67.6%)**	**0.21**	4 (50.0%)	21 (72.4%)	0.39
Mucosal involvement	**8 (38.1%)**	**14 (37.8%)**	**1.00**	5 (62.5%)	9 (31.0%)	0.22
Neurological	**4 (4.8%)**	**3 (1.3%)**	**0.078**	0 (0.0%)	3 (1.5%)	1.00
Gastrointestinal	**1 (1.2%)**	**5 (2.1%)**	**1.00**	3 (8.1%)	2 (1.0%)	0.029
Other	**2 (2.4%)**	**1 (0.4%)**	**0.17**	0 (0.0%)	1 (0.5%)	1.00
***Antibiotic treatment after presentation***						
Total	**73 (89.0%)**	**184 (79.7%)**	**0.065**	31 (83.8%)	153 (78.9%)	0.66
Amoxicillin ± clavulanic acid	**37 (45.1%)**	**31 (13.4%)**	** < 0.0001**	3 (8.1%)	28 (14.4%)	0.43
Macrolides	**30 (36.6%)**	**102 (44.2%)**	**0.24**	11 (29.7%)	91 (46.9%)	0.07
Doxycycline	**24 (29.3%)**	**78 (33.8%)**	**0.49**	18 (48.6%)	60 (30.9%)	0.056
Other	**7 (8.5%)**	**9 (3.9%)**	**0.14**	4 (10.8%)	5 (2.6%)	0.039
NA	**1**	**5**		0	5	
***Steroid treatment after presentation***						
Total	**8 (10.1%)**	**55 (23.9%)**	**0.0092**	7 (20.0%)	48 (24.6%)	0.67
NA	**4**	**6**		2	4	
***Outcome***						
Hospitalization	**36 (43.9%)**	**91 (38.6%)**	**0.43**	17 (45.9%)	74 (37.2%)	0.36
NA	**1**	**0**		0	0	
LOS (days)	**4.0 (3.0, 6.0)**	**4.0 (2.0, 5.0)**	**0.47**	3.5 (3.0, 6.0)	4.0 (2.0, 5.0)	0.83
NA	**1**	**4**		3	1	
ICU	**4 (4.9%)**	**12 (5.1%)**	**1.00**	3 (8.1%)	9 (4.5%)	0.41
NA	**1**	**0**		0	0	
Long-term sequelae^c^	**4 (6.0%)**	**4 (6.3%)**	**1.00**	1 (6.7%)	3 (6.3%)	1.00
NA	**16**	**173**		22	151	

More detailed information on parameters or subgroups can be found in Tables S1–S10. The annual figures always refer to the 12-month period from April 1 to March 31 (e.g., April 1, 2015–March 31, 2016). Continuous variables are summarized as median (1st quartile, 3rd quartile), categorical variables as no. (%) or no. *P* values were calculated by the Kruskal–Wallis rank sum test (continuous variables) or Fisher’s exact test (categorical variables). Abbreviations: *ICU* intensive care unit, *LOS* length of stay, *LRTI* lower respiratory tract infection, *NA* not available, *NPI* non-pharmaceutical intervention, *PCR* polymerase chain reaction, *RTI* respiratory tract infection, *URTI* upper respiratory tract infection

^a^Symptoms within ± 30 days of the patient’s symptom onset

^b^Chest radiographs originating from an earlier presentation were excluded

^c^Pre-NPI: respiratory (*n* = 2), neurological (*n* = 1), and other or not specified (*n* = 1). Post-NPI: respiratory (*n* = 1), dermatological/other (*n* = 1), gastrointestinal (*n* = 1), and respiratory/dermatological/gastrointestinal/other (*n* = 1). The following diagnoses were not considered long-term sequelae: one patient was later diagnosed with juvenile idiopathic arthritis (pre-NPI), one patient was later diagnosed with perityphlitic abscess (post-NPI), and one with postural orthostatic tachycardia syndrome (POTS) and gastritis (post-NPI)

Laboratory analyses showed lower neutrophil (median 6.06 vs 8.40 G/L) and platelet (median 294 vs 349 G/L) counts post-NPI than pre-NPI, while C-reactive protein levels did not differ between periods (median 22 vs 25 mg/L; Table S8). Rhinovirus was the most common co-detected pathogen and was found more frequently post-NPI than pre-NPI (28.0% vs 6.0%; Table S9).

Pulmonary infiltrates were detected with a similar frequency in both periods (85.7% vs 84.2%; Table 1). Notably, pleural effusions were observed more frequently in chest radiographs post-NPI than pre-NPI (45.7% vs 28.9%).

The diagnosis of CAP has decreased (72.9% vs 92.8%) and that of upper respiratory tract (URT) infection has increased (14.4% vs 1.2%) post-NPI compared to pre-NPI (Table 1). A separate analysis of patients with lower respiratory tract infections in the pre- and post-NPI period is shown in Table S10.

Interestingly, more obstructive diseases were observed post-NPI compared to pre-NPI (18.6% vs 9.6%). Overall, extrapulmonary manifestations occurred less frequently post-NPI than pre-NPI (18.6% vs 30.1%; Table 1 and Table S4), particularly dermatological (15.7% vs 25.3%; Tables S5–S6) and neurological (1.3% vs 4.8%) manifestations.

Antibiotic treatment at presentation was initiated less frequently post-NPI than pre-NPI (79.7% vs 89.0%; Table 1). In contrast, corticosteroid treatment increased post-NPI compared to pre-NPI (23.9% vs 10.1%; Table S2).

Hospitalization rate (38.6% vs 43.9%) and length of stay (median, 4 [IQR, 2–5] vs 4 [IQR, 3–6] days) were similar post-NPI and pre-NPI (Table 1), while generalized linear models showed a trend toward fewer hospitalizations post-NPI (odds ratio [OR], 0.72 [95% CI, 0.42–1.23]; *P* = 0.22; Tables S11–S14). The same was observed for ICU admission rate (5.1% post-NPI vs 4.9% pre-NPI), with a trend toward fewer ICU admissions post-NPI (OR, 0.90 [95% CI, 0.29–3.34]; *P* = 0.86; Tables S15–S18). Long-term sequelae were reported equally between periods (6.3% post-NPI vs 6.0% pre-NPI; Table 1), and no deaths were observed during any period (Table S2).

## Discussion

This retrospective, comparative cohort study of *M. pneumoniae* infections in children suggests a possible shift in clinical features of re-emerging infections compared to the pre-COVID-19 pandemic period. However, there is currently no clear indication of a significant increase in overall disease severity.

The near-complete absence of *M. pneumoniae* for more than three years has raised major concerns about the risk of large outbreaks and potential changes in clinical phenotype and disease severity of infections due to waning herd immunity [7]. In fact, disease outbreaks due to the global re-emergence of *M. pneumoniae* were even higher than expected [1, 2, 5, 8, 9]. We were able to evaluate the clinical phenotype and disease severity of re-emerging *M. pneumoniae* infections using our unique cohort as a reference [4] and observed the following clinically relevant changes: (i) shift in the age distribution toward older children (as reported by others [10–12]); (ii) more frequent obstructive disease; (iii) more frequent pleural effusion in chest radiograph, presenting with chest pain (indicating a more pronounced pulmonary inflammatory response [13]); and (iv) less frequent extrapulmonary manifestations.

There have been reports of increasing numbers of clinically severe disease in *M. pneumoniae*-infected children [14–16]. However, despite the exceptionally large wave of re-emerging *M. pneumoniae* infections, no statistically increased proportion of severe or worse outcomes could be observed globally compared with pre-COVID-19 pandemic *M. pneumoniae* epidemics [1, 17]. We even observed a trend toward a reduction in hospitalization and ICU admission rates after COVID-19 pandemic restrictions, despite *M. pneumoniae* infections being observed more frequently in patients with underlying diseases. The observation that there was no clear indication of a significant increase in overall disease severity was also corroborated by other reports [1, 10–12, 18, 19].

There are several limitations to our study. First, the patient cohort is geographically confined to the region of Zurich, Switzerland. The epidemiology and clinical manifestations may vary depending on the geographical region [1, 20]. Second, increased awareness due to the re-emergence may have led to increased testing for *M. pneumoniae* [1]. However, the “targeted” testing strategy—testing only children suspected to have *M. pneumoniae* CAP [4]—did not change between the periods. Third, *M. pneumoniae*-specific testing with singleplex PCR has been replaced by multiplex PCR as of October 12, 2020 [1]. This could have led to an increase in co-detections with *M. pneumoniae* and could explain why more URT infections (for which the clinical picture is not a testing indication) were diagnosed in association with *M. pneumoniae*. Fourth, we cannot say with certainty whether post-NPI detections were *M. pneumoniae* infection or carriage. This differentiation can be made using the ASC ELISpot assay [21], which was used to confirm *M. pneumoniae* infections in the pre-NPI period in the myCAP cohort [4]. Finally, macrolide-resistant *M. pneumoniae* (MR*Mp*), which can be associated with more severe disease and extrapulmonary manifestations [2], was tested only on request from a physician in case of clinically suspected MR*Mp* infection [1]. However, the local pre-NPI MR*Mp* rate in children was very low (2%) [22], and global data showed no increase in MR*Mp* rates during the re-emergence [1].

Further research within the global framework of the ESGMAC MAPS study [1] will determine if and how *M. pneumoniae* strain differences contribute to these phenotypic differences. Given the high number of cases, the observed trend of decreasing disease severity, and the overall increase in MR*Mp* worldwide due to increased global antibiotic prescribing, further studies on the efficacy of antibiotics in treating *M. pneumoniae* infections are urgently needed [23].

In summary, the clinical characteristics of *M. pneumoniae* infections have partly changed following COVID-19 pandemic restrictions, with evolving features such as obstructive phenotypes and pleural effusions accompanied by chest pain. Concerns about a more severe disease course of re-emerging infections were not confirmed. However, the observed changes in the clinical characteristics of *M. pneumoniae* infections highlight the need for continuous monitoring of these clinical phenotypes.

## Supplementary Information

Below is the link to the electronic supplementary material.

Supplementary file1 (PDF 470 KB)

## Data Availability

E.R. and P.M.M.S. have full access to all of the data in the study and take responsibility for the integrity of the data and the accuracy of the data analysis. Anonymized data will be made available upon reasonable request to the corresponding author.
