# Supplementary material for: Evolving clinical features of Mycoplasma pneumoniae infections following COVID-19 pandemic restrictions: a retrospective, comparative cohort study
Source: Eur J Pediatr. 2025 Aug 7;184(8):535. doi: 10.1007/s00431-025-06326-y (PMC12328521; doi:10.1007/s00431-025-06326-y)
Supplement: Supplementary file 1 — Supplementary file1 (PDF 470 KB) [file 431_2025_6326_MOESM1_ESM.pdf]

Supplementary material

Supplementary Figure S1 Study flow

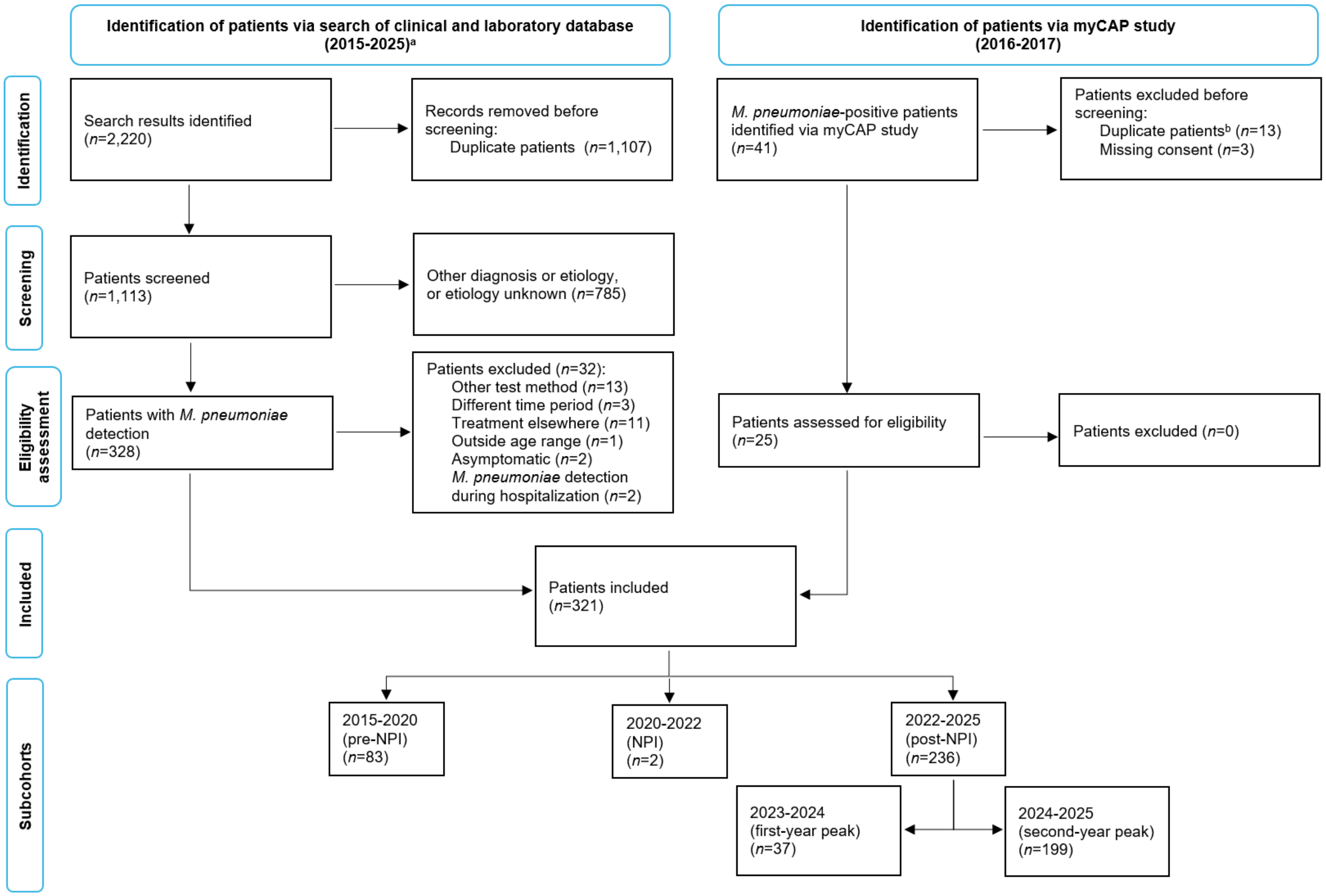

The annual figures always refer to the 12-month period April 1–March 31 (e.g., April 1, 2015–March 31, 2016). **Abbreviations:** NPI, non-pharmaceutical intervention.

[a] The search of clinical and laboratory database was retrospectively performed as follows: (1) Electronic laboratory records were systematically reviewed by an IT-

specialist at the hospital for positive *M. pneumoniae*-specific polymerase chain reaction (PCR) tests from April 1, 2015 to March 31, 2025. Only patients with a signed consent were included. (2) Additionally, medical records were systematically reviewed by an IT-specialist at the hospital from April 1, 2015 to March 31, 2025 using the search terms '*Mycoplasma pneumoniae*', '*M. pneumoniae'*, 'Mykoplasmen', 'Mycoplasmen' (german for mycoplasma), '*Mykoplasma pneumoniae*', 'Atypische Pneumonie' (german for atypical pneumonia). Only patients with a signed consent were included.

[b] 13 patients who were identified in the search of clinical and laboratory databases (on the left) had also participated in the myCAP study (BASEC no. 2016-00148) [4].

Supplementary Table S1 Supplementary demographic characteristics of children with *Mycoplasma pneumoniae* detection by PCR from April 1, 2015 to March 31, 2025

Variable	2015-2020 (pre-NPI) (*n* = 83)	2022-2025 (post-NPI) (*n* = 236)	p value	2023-2024 (post-NPI first-year peak) (*n* = 37)	2024-2025 (post-NPI second-year peak) (*n* = 199)	p value
Epidemiology						
Season at presentation			**0.0039**			< 0.0001
Spring (March-May)	**14 (16.9%)**	**13 (5.5%)**		3 (8.1%)	10 (5.0%)	
Summer (June-August)	**17 (20.5%)**	**75 (31.8%)**		1 (2.7%)	74 (37.2%)	
Autumn (September-November)	**36 (43.4%)**	**117 (49.6%)**		22 (59.5%)	95 (47.7%)	
Winter (December-February)	**16 (19.3%)**	**31 (13.1%)**		11 (29.7%)	20 (10.1%)	
Underlying diseases						
Any underlying diseases	**11 (13.3%)**	**63 (26.7%)**	**0.015**	11 (29.7%)	52 (26.1%)	0.69
Asthma	**4 (4.8%)**	**15 (6.4%)**	**0.79**	1 (2.7%)	14 (7.0%)	0.48
Other pulmonary	**2 (2.4%)**	**6 (2.5%)**	**1.00**	1 (2.7%)	5 (2.5%)	1.00
Cardiovascular	**1 (1.2%)**	**13 (5.5%)**	**0.13**	3 (8.1%)	10 (5.0%)	0.44
Gastrointestinal	**3 (3.6%)**	**8 (3.4%)**	**1.00**	2 (5.4%)	6 (3.0%)	0.61
Neurological	**2 (2.4%)**	**13 (5.5%)**	**0.37**	4 (10.8%)	9 (4.5%)	0.13
Primary or secondary immunodeficiency	**0 (0.0%)**	**6 (2.5%)**	**0.35**	2 (5.4%)	4 (2.0%)	0.24
Other	**6 (7.2%)**	**41 (17.4%)**	**0.03**	7 (18.9%)	34 (17.1%)	0.81
***Family members with RTI*[a]**						
Any family members with RTI	**47 (71.2%)**	**107 (74.8%)**	**0.61**	10 (71.4%)	97 (75.2%)	0.75
Father	**18 (27.3%)**	**31 (21.7%)**	**0.38**	2 (14.3%)	29 (22.5%)	0.73
Mother	**26 (39.4%)**	**31 (21.7%)**	**0.012**	3 (21.4%)	28 (21.7%)	1.00
Sibling	**32 (48.5%)**	**56 (39.2%)**	**0.23**	7 (50.0%)	49 (38.0%)	0.40
NA	**17**	**93**		23	70	
Prior antibiotic treatment						
Total	**38 (45.8%)**	**65 (27.5%)**	**0.0027**	17 (45.9%)	48 (24.1%)	0.009
Amoxicillin ± clavulanic acid	**28 (33.7%)**	**41 (17.4%)**	**0.003**	14 (37.8%)	27 (13.6%)	0.0014
Macrolide	**4 (4.8%)**	**28 (11.9%)**	**0.088**	5 (13.5%)	23 (11.6%)	0.78
Other	**8 (9.6%)**	**7 (3.0%)**	**0.029**	5 (13.5%)	2 (1.0%)	0.0012
Agent unknown	**0 (0.0%)**	**1 (0.4%)**	**1.00**	0 (0.0%)	1 (0.5%)	1.00
Prior corticosteroid treatment						
Total	**4 (6.6%)**	**30 (12.7%)**	**0.26**	4 (10.8%)	26 (13.1%)	1.00
Systemic	**3 (4.9%)**	**14 (5.9%)**	**1.00**	3 (8.1%)	11 (5.5%)	0.47
Inhaled	**1 (1.6%)**	**16 (6.8%)**	**0.21**	1 (2.7%)	15 (7.5%)	0.48
Other or application form unknown	**0 (0.0%)**	**4 (1.7%)**	**0.58**	1 (2.7%)	3 (1.5%)	0.50
NA	**22**	**0**		0	0	

The annual figures always refer to the 12-month period April 1 to March 31 (e.g., April 1, 2015–March 31, 2016). Variables are summarized as no. (%) or no. P values were calculated by the Fisher's exact test. **Abbreviations:** NA, not available; NPI, non-pharmaceutical intervention; PCR, polymerase chain reaction; RTI, respiratory tract infection.

[a] Symptoms within ± 30 days of the patient's symptom onset.

Supplementary Table S2 Supplementary clinical characteristics of children with *Mycoplasma pneumoniae* detection by PCR from April 1, 2015 to March 31, 2025

Variable	2015-2020 (pre-NPI) (*n* = 83)	2022-2025 (post-NPI) (*n* = 236)	p value	2023-2024 (post-NPI first-year peak) (*n* = 37)	2024-2025 (post-NPI second-year peak) (*n* = 199)	p value
Symptoms at presentation						
Rhinitis	**22 (26.5%)**	**66 (28.1%)**	**0.89**	12 (32.4%)	54 (27.3%)	0.55
Sore throat	**16 (19.3%)**	**21 (8.9%)**	**0.016**	7 (18.9%)	14 (7.1%)	0.03
Cough	**74 (89.2%)**	**221 (94.0%)**	**0.15**	36 (97.3%)	185 (93.4%)	0.70
Wheezing	**2 (2.4%)**	**3 (1.3%)**	**0.61**	0 (0.0%)	3 (1.5%)	1.00
Chest pain	**2 (2.4%)**	**21 (8.9%)**	**0.05**	7 (18.9%)	14 (7.1%)	0.03
Headache	**6 (7.2%)**	**18 (7.7%)**	**1.00**	3 (8.1%)	15 (7.6%)	1.00
Ear pain	**3 (3.6%)**	**10 (4.3%)**	**1.00**	3 (8.1%)	7 (3.5%)	0.20
Abdominal pain	**9 (10.8%)**	**26 (11.1%)**	**1.00**	5 (13.5%)	21 (10.6%)	0.57
Nausea and/or vomiting	**16 (19.3%)**	**59 (25.1%)**	**0.37**	14 (37.8%)	45 (22.7%)	0.063
Diarrhea	**2 (2.4%)**	**20 (8.5%)**	**0.077**	6 (16.2%)	14 (7.1%)	0.10
NA	**0**	**1**		0	1	
Prodromal symptom duration (days)	**9.0 (6.0,11.0)**	**7.0 (5.0,10.0)**	**0.14**	10.0 (6.0,14.0)	7.0 (5.0,10.0)	0.044
NA	**1**	**5**		0	5	
Vital signs at presentation						
Fever	**38 (50.0%)**	**64 (28.2%)**	**0.00072**	7 (19.4%)	57 (29.8%)	0.23
NA	**7**	**9**		1	8	
Fever (°C)	**39.0 (38.5,39.4)**	**38.6 (38.1,38.8)**	**0.00014**	39.2 (38.5,39.7)	38.5 (38.1,38.8)	0.033
Oxygen saturation <93%	**21 (35.6%)**	**64 (28.1%)**	**0.27**	12 (33.3%)	52 (27.1%)	0.43
NA	**24**	**8**		1	7	
Tachypnea	**62 (86.1%)**	**130 (85.0%)**	**1.00**	15 (78.9%)	115 (85.8%)	0.49
NA	**11**	**83**		18	65	
Dyspnea	**22 (26.5%)**	**66 (28.1%)**	**0.89**	10 (27.0%)	56 (28.3%)	1.00
NA	**0**	**1**		0	1	
Cyanosis	**1 (1.2%)**	**1 (0.4%)**	**0.46**	0 (0.0%)	1 (0.5%)	NA
NA	**0**	**4**		0	4	
Other signs at presentation						
Grunting	**0 (0.0%)**	**3 (1.3%)**	**0.57**	0 (0.0%)	3 (1.5%)	1.00
Nasal flaring	**5 (6.0%)**	**8 (3.4%)**	**0.34**	1 (2.7%)	7 (3.6%)	1.00
Retractions	**25 (30.1%)**	**56 (24.1%)**	**0.31**	6 (16.2%)	50 (25.6%)	0.30
Abnormal auscultatory lung findings	**63 (75.9%)**	**161 (69.4%)**	**0.32**	26 (70.3%)	135 (69.2%)	1.00
Pharyngitis/Tonsillitis	**34 (41.0%)**	**104 (44.8%)**	**0.61**	18 (48.6%)	86 (44.1%)	0.72
Otitis media	**6 (7.2%)**	**32 (13.8%)**	**0.17**	5 (13.5%)	27 (13.8%)	1.00
Enlarged lymph nodes	**15 (18.1%)**	**32 (13.8%)**	**0.37**	5 (13.5%)	27 (13.8%)	1.00
Dermatological findings	**18 (21.7%)**	**24 (10.3%)**	**0.014**	6 (16.2%)	18 (9.2%)	0.24
Dermatological prodrome (days)	**1.0 (0.0,2.0)**	**2.0 (0.0,5.0)**	**0.67**	5.0 (5.0,5.0)	1.5 (0.0,4.5)	0.43
NA	**7**	**15**		5	10	

**Variable**	**2015-2020 (pre-NPI) (*n* = 83)**	**2022-2025 (post-NPI) (*n* = 236)**	**p value**	**2023-2024 (post-NPI first-year peak) (*n* = 37)**	**2024-2025 (post-NPI second-year peak) (*n* = 199)**	**p value**
Neurological findings	**3 (3.6%)**	**8 (3.4%)**	**1.00**	1 (2.7%)	7 (3.6%)	1.00
Neurological prodrome (days)	**2.0 (0.0,10.0)**	**0.0 (0.0,1.0)**	**0.20**	0.0 (0.0,0.0)	0.0 (0.0,1.0)	0.53
NA	**0**	**1**		0	1	
NA	**0**	**4**		0	4	
Antibiotic treatment after presentation						
Total	**73 (89.0%)**	**184 (79.7%)**	**0.065**	31 (83.8%)	153 (78.9%)	0.66
Amoxicillin ± clavulanic acid	**37 (45.1%)**	**31 (13.4%)**	**< 0.0001**	3 (8.1%)	28 (14.4%)	0.43
Clarithromycin	**30 (36.6%)**	**99 (42.9%)**	**0.36**	10 (27.0%)	89 (45.9%)	0.045
Azithromycin	**0 (0.0%)**	**5 (2.2%)**	**0.33**	2 (5.4%)	3 (1.5%)	0.18
Doxycycline	**24 (29.3%)**	**78 (33.8%)**	**0.49**	18 (48.6%)	60 (30.9%)	0.056
Other	**7 (8.5%)**	**9 (3.9%)**	**0.14**	4 (10.8%)	5 (2.6%)	0.039
NA	**1**	**5**		0	5	
Steroid treatment after presentation						
Total	**8 (10.1%)**	**55 (23.9%)**	**0.0092**	7 (20.0%)	48 (24.6%)	0.67
Systemic	**6 (7.6%)**	**44 (19.1%)**	**0.02**	6 (17.1%)	38 (19.5%)	1.00
Inhaled	**0 (0.0%)**	**14 (6.1%)**	**0.025**	1 (2.9%)	13 (6.7%)	0.70
Other	**4 (5.1%)**	**5 (2.2%)**	**0.24**	1 (2.9%)	4 (2.1%)	0.57
NA	**4**	**6**		2	4	
***Long-term sequelae*[a]**						
Total	**4 (6.0%)**	**4 (6.3%)**	**1.00**	1 (6.7%)	3 (6.3%)	1.00
Death	**0 (0.0%)**	**0 (0.0%)**	**1.00**	0 (0.0%)	0 (0.0%)	1.00
Respiratory[b]	**2 (3.0%)**	**2 (3.2%)**	**1.00**	0 (0.0%)	2 (4.2%)	1.00
Dermatological[c]	**0 (0.0%)**	**2 (3.2%)**	**0.23**	1 (6.7%)	1 (2.1%)	0.42
Gastrointestinal[d]	**0 (0.0%)**	**2 (3.2%)**	**0.23**	0 (0.0%)	2 (4.2%)	1.00
Neurological[e]	**1 (1.5%)**	**0 (0.0%)**	**1.00**	0 (0.0%)	0 (0.0%)	1.00
Other or not specified[f]	**1 (1.5%)**	**2 (3.2%)**	**0.61**	1 (6.7%)	1 (2.1%)	0.42
NA	**16**	**173**		22	151	

The annual figures always refer to the 12-month period April 1 to March 31 (e.g., April 1, 2015–March 31, 2016). Continuous variables are summarized as median (1st quartile, 3rd quartile), categorical variables as no. (%) or no. P values were calculated by the Kruskal–Wallis rank sum test (continuous variables) or Fisher's exact test (categorical variables). **Abbreviations:** NA, not available; NPI, non-pharmaceutical intervention; PCR, polymerase chain reaction.

[a] Pre-NPI, one patient was later diagnosed with juvenile idiopathic arthritis. Post-NPI, one patient was later diagnosed with perityphlitic abscess, and one with postural orthostatic tachycardia syndrome (POTS) and gastritis. These diagnoses were <u>not</u> considered long-term sequelae.

[b] Pre-NPI: bronchiolitis obliterans (*n*=1), unspecific exertional dyspnea (*n*=1); post-NPI: unspecific cough (*n*=1), unspecific exertional dyspnea/cough/chest pain (*n*=1).

[c] Postinflammatory pigmentary alteration (*n*=1), mucosal inflammation (*n*=1).

[d] Colitis with stenosis (*n*=1), unspecific abdominal pain/nausea (*n*=1).

[e] Abnormal gait (*n*=1).

[f] Pre-NPI: not specified (*n*=1), post-NPI: reduced physical capacity (*n*=1), fatigue (*n*=1).

Supplementary Table S3 Supplementary radiographic findings of children with *Mycoplasma pneumoniae* detection by PCR from April 1, 2015 to March 31, 2025

Variable	2015-2020 (pre-NPI) (*n* = 77)	2022-2025 (post-NPI) (*n* = 141)	p value	2023-2024 (post-NPI first-year peak) (*n* = 30)	2024-2025 (post-NPI second-year peak) (*n* = 111)	p value
Radiographic findings[a]						
Pulmonary infiltrate in chest radiograph	**64 (84.2%)**	**120 (85.7%)**	**0.84**	24 (80.0%)	96 (87.3%)	0.38
Infiltrate type			**0.0041**			0.60
Alveolar (Consolidation)	**52 (81.3%)**	**114 (95.0%)**		24 (100.0%)	90 (93.8%)	
Interstitial	**12 (18.8%)**	**6 (5.0%)**		0 (0.0%)	6 (6.3%)	
Consolidation type			**0.89**			0.70
Single lobar infiltrate	**31 (59.6%)**	**65 (57.0%)**		12 (50.0%)	53 (58.9%)	
Multilobar infiltrates (unilateral)	**9 (17.3%)**	**19 (16.7%)**		5 (20.8%)	14 (15.6%)	
Multilobar infiltrates (bilateral)	**12 (23.1%)**	**30 (26.3%)**		7 (29.2%)	23 (25.6%)	
Interstitial infiltrate type			**0.33**			1.00
Reticular	**12 (100.0%)**	**5 (83.3%)**		0 (0.0%)	5 (83.3%)	
Nodular	**0 (0.0%)**	**1 (16.7%)**		0 (0.0%)	1 (16.7%)	
Pleural effusion	**22 (28.9%)**	**64 (45.7%)**	**0.02**	17 (56.7%)	47 (42.7%)	0.22
NA	**1**	**1**		0	1	

The annual figures always refer to the 12-month period April 1 to March 31 (e.g., April 1, 2015–March 31, 2016). Variables are summarized as no. (%) or no. P values were calculated by the Fisher's exact test. **Abbreviations:** NA, not available; NPI, non-pharmaceutical intervention; PCR, polymerase chain reaction.

[a] Chest radiographs originating from an earlier presentation were excluded.

Supplementary Table S4 Extrapulmonary manifestations of children with *Mycoplasma pneumoniae* detection by PCR from April 1, 2015 to March 31, 2025

**Variable**	**2015-2020 (pre-NPI) (*n* = 25)**	**2022-2025 (post-NPI) (*n* = 44)**	**p value**	**2023-2024 (post-NPI first-year peak) (*n* = 11)**	**2024-2025 (post-NPI second-year peak) (*n* = 33)**	**p value**
Extrapulmonary manifestations						
Dermatological	**21 (84.0%)**	**37 (84.1%)**	**1.00**	8 (72.7%)	29 (87.9%)	0.34
MIRM/RIME	**4 (19.0%)**	**3 (8.1%)**	**0.24**	1 (12.5%)	2 (6.9%)	0.53
Urticaria	**7 (33.3%)**	**9 (24.3%)**	**0.55**	0 (0.0%)	9 (31.0%)	0.16
Other	**10 (47.6%)**	**25 (67.6%)**	**0.17**	7 (87.5%)	18 (62.1%)	0.23
Neurological	**4 (16.0%)**	**3 (6.8%)**	**0.25**	0 (0.0%)	3 (9.1%)	0.56
GBS	**1 (25.0%)**	**0 (0.0%)**	**1.00**	0 (NA%)	0 (0.0%)	1.00
Meningitis/Meningeal Irritation	**3 (75.0%)**	**3 (100.0%)**	**1.00**	0 (NA%)	3 (100.0%)	1.00
Gastrointestinal	**1 (4.0%)**	**5 (11.4%)**	**0.41**	3 (27.3%)	2 (6.1%)	0.091
Hepatitis	**1 (100.0%)**	**1 (20.0%)**	**0.33**	1 (33.3%)	0 (0.0%)	1.00
Gastritis/Enteritis/Colitis	**0 (0.0%)**	**4 (80.0%)**	**0.33**	2 (66.7%)	2 (100.0%)	1.00
Other	**2 (8.0%)**	**1 (2.3%)**	**0.30**	0 (0.0%)	1 (3.0%)	NA

The annual figures always refer to the 12-month period April 1 to March 31 (e.g., April 1, 2015–March 31, 2016). Variables are summarized as no. (%). P values were calculated by the Fisher's exact test. **Abbreviations:** GBS, Guillain-Barré syndrome; MIRM, *Mycoplasma pneumoniae*-induced rash and mucositis; NA, not available; NPI, non-pharmaceutical intervention; PCR, polymerase chain reaction; RIME, reactive infectious mucocutaneous eruption.

Supplementary Table S5 Supplementary dermatological findings of children with *Mycoplasma pneumoniae* detection by PCR from April 1, 2015 to March 31, 2025 with cutaneous involvement

Variable	2015-2020 (pre-NPI) (*n* = 18)	2022-2025 (post-NPI) (*n* = 25)	p value	2023-2024 (post-NPI first-year peak) (*n* = 4)	2024-2025 (post-NPI second-year peak) (*n* = 21)	p value
Cutaneous involvement						
Location			**0.94**			0.098
Acral	**6 (42.9%)**	**5 (27.8%)**		2 (50.0%)	3 (21.4%)	
Truncal	**4 (28.6%)**	**7 (38.9%)**		0 (0.0%)	7 (50.0%)	
Face	**0 (0.0%)**	**1 (5.6%)**		1 (25.0%)	0 (0.0%)	
Generalized	**4 (28.6%)**	**5 (27.8%)**		1 (25.0%)	4 (28.6%)	
NA	**4**	**7**		0	7	
Morphological characteristics[a]						
Macular	**7 (43.8%)**	**14 (58.3%)**	**0.52**	4 (100.0%)	10 (50.0%)	0.11
Urticarial	**7 (43.8%)**	**9 (37.5%)**	**0.75**	0 (0.0%)	9 (45.0%)	0.26
Papular	**4 (25.0%)**	**4 (16.7%)**	**0.69**	0 (0.0%)	4 (20.0%)	1.00
Targetoid	**4 (25.0%)**	**1 (4.2%)**	**0.14**	1 (25.0%)	0 (0.0%)	0.17
Vesiculobullous	**2 (12.5%)**	**1 (4.2%)**	**0.55**	1 (25.0%)	0 (0.0%)	0.17
Other	**3 (18.8%)**	**1 (4.2%)**	**0.28**	0 (0.0%)	1 (5.0%)	1.00
NA	**2**	**1**		0	1	

The annual figures always refer to the 12-month period April 1 to March 31 (e.g., April 1, 2015–March 31, 2016). Variables are summarized as no. (%) or no. P values were calculated by the Fisher's exact test. **Abbreviations:** NA, not available; NPI, non-pharmaceutical intervention; PCR, polymerase chain reaction.

[a] Pre-NPI: macular/urticarial (*n*=1), macular/papular (*n*=2), macular/targetoid (*n*=1), macular/other (*n*=1), urticarial/papular/targetoid (*n*=1), urticarial/papular/targetoid/other (*n*=1), targetoid/vesiculobullous (*n*=1); post-NPI: macular/papular (*n*=2), macular/papular/other (*n*=1), macular/targetoid/vesiculobullous (*n*=1).

Supplementary Table S6 Supplementary dermatological findings of children with *Mycoplasma pneumoniae* detection by PCR from April 1, 2015 to March 31, 2025 with mucosal involvement

**Variable**	**2015-2020 (pre-NPI) (*n* = 8)**	**2022-2025 (post-NPI) (*n* = 14)**	**p value**	**2023-2024 (post-NPI first-year peak) (*n* = 5)**	**2024-2025 (post-NPI second-year peak) (*n* = 9)**	**p value**
Mucosal involvement						
Ocular	**7 (87.5%)**	**9 (64.3%)**	**0.35**	2 (40.0%)	7 (77.8%)	0.27
Oral	**6 (75.0%)**	**8 (57.1%)**	**0.65**	4 (80.0%)	4 (44.4%)	0.30
Urogenital	**3 (37.5%)**	**2 (14.3%)**	**0.31**	1 (20.0%)	1 (11.1%)	1.00
Anal	**1 (12.5%)**	**2 (14.3%)**	**1.00**	1 (20.0%)	1 (11.1%)	1.00

The annual figures always refer to the 12-month period April 1 to March 31 (e.g., April 1, 2015–March 31, 2016). Variables are summarized as no. (%). P values were calculated by the Fisher's exact test. **Abbreviations:** NPI, non-pharmaceutical intervention; PCR, polymerase chain reaction.

Supplementary Table S7 Hospitalization reason of hospitalized children with *Mycoplasma pneumoniae* detection by PCR from April 1, 2015 to March 31, 2025

**Variable**	**2015-2020 (pre-NPI) (*n* = 36)**	**2022-2025 (post-NPI) (*n* = 91)**	**p value**	**2023-2024 (post-NPI first-year peak) (*n* = 17)**	**2024-2025 (post-NPI second-year peak) (*n* = 74)**	**p value**
Reason for hospitalization						
Additional oxygen demand	**22 (62.9%)**	**79 (86.8%)**	**0.0051**	15 (88.2%)	64 (86.5%)	1.00
Extrapulmonary manifestation	**7 (20.0%)**	**5 (5.5%)**	**0.02**	2 (11.8%)	3 (4.1%)	0.23
Other	**6 (17.1%)**	**7 (7.7%)**	**0.19**	0 (0.0%)	7 (9.5%)	0.34
NA	**1**	**0**		0	0	

The annual figures always refer to the 12-month period April 1 to March 31 (e.g., April 1, 2015–March 31, 2016). Variables are summarized as no. (%) or no. P values were calculated by the Fisher's exact test. **Abbreviations:** NA, not available; NPI, non-pharmaceutical intervention; PCR, polymerase chain reaction.

Supplementary Table S8 Laboratory findings of children with *Mycoplasma pneumoniae* detection by PCR from April 1, 2015 to March 31, 2025

Variable	2015-2020 (pre-NPI) (*n* = 83)	2022-2025 (post-NPI) (*n* = 236)	p value	2023-2024 (post-NPI first-year peak) (*n* = 37)	2024-2025 (post-NPI second-year peak) (*n* = 199)	p value
Leucocytes (G/L)	**11.25 (7.70,13.95)**	**9.20 (7.19,12.77)**	**0.11**	9.42 (6.51,12.77)	8.82 (7.36,12.72)	0.84
NA	**29**	**137**		10	127	
Neutrophiles (G/L)	**8.40 (4.68,10.23)**	**6.06 (4.12,9.27)**	**0.044**	6.27 (3.74,8.76)	6.04 (4.19,9.60)	0.55
NA	**34**	**148**		12	136	
Eosinophils (G/L)	**0.12 (0.05,0.26)**	**0.10 (0.03,0.21)**	**0.20**	0.10 (0.03,0.25)	0.10 (0.02,0.20)	0.88
NA	**38**	**161**		15	146	
Basophils (G/L)	**0.04 (0.03,0.07)**	**0.03 (0.02,0.04)**	**0.027**	0.03 (0.02,0.05)	0.03 (0.02,0.04)	0.44
NA	**51**	**161**		15	146	
Lymphocytes (G/L)	**2.09 (1.40,2.68)**	**1.78 (1.28,2.36)**	**0.17**	1.83 (1.28,2.56)	1.77 (1.24,2.28)	0.75
NA	**33**	**143**		11	132	
Monocytes (G/L)	**0.77 (0.50,1.03)**	**0.62 (0.46,0.87)**	**0.06**	0.62 (0.41,0.88)	0.62 (0.49,0.86)	0.64
NA	**33**	**148**		13	135	
Hemoglobin (g/L)	**130 (121,137)**	**126 (117,134)**	**0.11**	127 (118,135)	125 (117,133)	0.61
NA	**30**	**141**		11	130	
Platelets (G/L)	**349 (288,431)**	**294 (244,371)**	**0.0066**	294 (246,383)	294 (237,369)	0.78
NA	**31**	**144**		11	133	
C-reactive protein (mg/L)	**25 (10,63)**	**22 (9,48)**	**0.41**	25 (9,66)	17 (8,39)	0.21
NA	**32**	**137**		10	127	

The annual figures always refer to the 12-month period April 1 to March 31 (e.g., April 1, 2015–March 31, 2016). Laboratory values in the range of ±1 day to the presentation date were included in the analysis. Variables are summarized as median (1st quartile, 3rd quartile), missing variables as no. P values were calculated by the Kruskal–Wallis rank sum test. **Abbreviations:** NA, not available; NPI, non-pharmaceutical intervention; PCR, polymerase chain reaction.

Supplementary Table S9 Co-detection of other bacteria and/or viruses in the respiratory tract of children with *Mycoplasma pneumoniae* detection by PCR from April 1, 2015 to March 31, 2025

**Variable**	**2015-2020 (pre-NPI) (*n* = 83)**	**2022-2025 (post-NPI) (*n* = 236)**	**p value**	**2023-2024 (post-NPI first-year peak) (*n* = 37)**	**2024-2025 (post-NPI second-year peak) (*n* = 199)**	**p value**
Total	**14 (16.9%)**	**90 (38.1%)**	**0.00036**	12 (32.4%)	78 (39.2%)	0.47
Bacterial co-detection[a]						
Total	**2 (2.4%)**	**12 (5.1%)**	**0.53**	3 (8.1%)	9 (4.5%)	0.41
Chlamydia pneumoniae	**1 (1.2%)**	**4 (1.7%)**	**1.00**	2 (5.4%)	2 (1.0%)	0.12
Bordetella pertussis	**0 (0.0%)**	**3 (1.3%)**	**0.57**	0 (0.0%)	3 (1.5%)	1.00
Bordetella parapertussis	**0 (0.0%)**	**0 (0.0%)**	**1.00**	0 (0.0%)	0 (0.0%)	1.00
Other[b]	**1 (1.2%)**	**5 (2.1%)**	**1.00**	1 (2.7%)	4 (2.0%)	0.58
Viral co-detection						
Total	**13 (15.7%)**	**87 (36.9%)**	**0.00031**	10 (27.0%)	77 (38.7%)	0.20
Influenza virus	**0 (0.0%)**	**3 (1.3%)**	**0.57**	0 (0.0%)	3 (1.5%)	1.00
A	**0**	**2**	**1.00**	0	2	1.00
B	**0**	**0**	**1.00**	0	0	1.00
RSV	**2 (2.4%)**	**7 (3.0%)**	**1.00**	0 (0.0%)	7 (3.5%)	0.60
Coronavirus	**1 (1.2%)**	**12 (5.1%)**	**0.20**	2 (5.4%)	10 (5.0%)	1.00
229E	**0**	**0**	**1.00**	0	0	1.00
HKU1	**0**	**1**	**1.00**	1	0	0.16
NL63	**0**	**3**	**0.57**	0	3	1.00
OC43	**1**	**1**	**0.45**	0	1	1.00
MERS	**0**	**0**	**1.00**	0	0	1.00
SARS	**0**	**8**	**0.12**	1	7	1.00
Adenovirus	**6 (7.2%)**	**8 (3.4%)**	**0.21**	0 (0.0%)	8 (4.0%)	0.36
Human Metapneumovirus	**0 (0.0%)**	**0 (0.0%)**	**1.00**	0 (0.0%)	0 (0.0%)	1.00
Human Rhinovirus/Enterovirus	**5 (6.0%)**	**66 (28.0%)**	**< 0.0001**	8 (21.6%)	58 (29.1%)	0.43
Parainfluenza virus	**0 (0.0%)**	**13 (5.5%)**	**0.024**	2 (5.4%)	11 (5.5%)	1.00
1	**0**	**1**	**1.00**	0	1	1.00
2	**0**	**7**	**0.20**	0	7	0.60
3	**0**	**4**	**0.58**	2	2	0.12
4	**0**	**2**	**1.00**	0	2	1.00
Bocavirus	**2 (2.4%)**	**0 (0.0%)**	**0.067**	0 (0.0%)	0 (0.0%)	1.00

The annual figures always refer to the 12-month period April 1 to March 31 (e.g., April 1, 2015–March 31, 2016). Variables are summarized as no. (%) or no. P values were calculated by the Fisher's exact test. *M. pneumoniae*-specific testing with singleplex PCR has been replaced by multiplex PCR as of October 12, 2020 (supplementary appendix 3 in [1], page 16). **Abbreviations:** NPI, non-pharmaceutical intervention; PCR, polymerase chain reaction.

a For patients pre-NPI who had participated in the myCAP study, singleplex *Streptococcus pneumoniae*-specific PCR results performed as part of that study were excluded from the analyses to avoid testing bias.

b Pre-NPI: *Staphylococcus aureus* / *Haemophilus influenzae* (*n*=1); post-NPI: *Streptococcus pneumoniae* (*n*=1), *Haemophilus parainfluenzae* / *Rothia mucilaginosa* / *α-hemolytic streptococci* (*n*=1), *Streptococcus pneumoniae* / *Haemophilus influenzae* / *Acinteobacter junii* (*n*=1), *Haemophilus influenzae* (*n*=1), *Staphylococcus aureus* (*n*=1).

Supplementary Table S10 Demographic and clinical characteristics, and co-detection of other bacteria and/or viruses in the respiratory tract of children with lower respiratory tract infection and *Mycoplasma pneumoniae* detection by PCR from April 1, 2015 to March 31, 2025

Variable	2015-2020 (pre-NPI) (*n* = 81)	2022-2025 (post-NPI) (*n* = 199)	p value	2023-2024 (post-NPI first-year peak) (*n* = 35)	2024-2025 (post-NPI second-year peak) (*n* = 164)	p value
**DEMOGRAPHIC CHARACTERISTICS**						
Age (years)	**8.20 (5.60,10.20)**	**9.00 (5.30,11.40)**	**0.55**	11.00 (7.10,13.50)	8.45 (4.95,11.00)	0.00092
Sex, female	**32 (39.5%)**	**88 (44.2%)**	**0.51**	17 (48.6%)	71 (43.3%)	0.58
Underlying diseases	**11 (13.6%)**	**51 (25.6%)**	**0.027**	9 (25.7%)	42 (25.6%)	1.00
Family members with RTI[a]	**46 (71.9%)**	**85 (71.4%)**	**1.00**	10 (76.9%)	75 (70.8%)	0.76
NA	**17**	**80**		22	58	
**CLINICAL CHARACTERISTICS**						
Symptoms at presentation						
Rhinitis	**22 (27.2%)**	**47 (23.7%)**	**0.54**	11 (31.4%)	36 (22.1%)	0.27
Sore throat	**16 (19.8%)**	**18 (9.1%)**	**0.025**	7 (20.0%)	11 (6.7%)	0.022
Cough	**73 (90.1%)**	**187 (94.4%)**	**0.20**	35 (100.0%)	152 (93.3%)	0.22
Wheezing	**2 (2.5%)**	**3 (1.5%)**	**0.63**	0 (0.0%)	3 (1.8%)	1.00
Chest pain	**2 (2.5%)**	**21 (10.6%)**	**0.029**	7 (20.0%)	14 (8.6%)	0.066
Headache	**6 (7.4%)**	**16 (8.1%)**	**1.00**	3 (8.6%)	13 (8.0%)	1.00
Ear pain	**3 (3.7%)**	**9 (4.5%)**	**1.00**	3 (8.6%)	6 (3.7%)	0.20
Abdominal pain	**9 (11.1%)**	**22 (11.1%)**	**1.00**	4 (11.4%)	18 (11.0%)	1.00
Nausea and/or vomiting	**16 (19.8%)**	**50 (25.3%)**	**0.36**	13 (37.1%)	37 (22.7%)	0.087
Diarrhea	**2 (2.5%)**	**17 (8.6%)**	**0.071**	5 (14.3%)	12 (7.4%)	0.19
NA	**0**	**1**		0	1	
Prodromal symptom duration (days)	**9.0 (6.0,11.0)**	**7.5 (6.0,10.5)**	**0.30**	10.0 (6.0,14.0)	7.0 (6.0,10.0)	0.072
NA	**0**	**3**		0	3	
Vital signs at presentation						
Fever	**38 (50.7%)**	**57 (29.4%)**	**0.0016**	7 (20.0%)	50 (31.4%)	0.22
NA	**6**	**5**		0	5	
Fever (°C)	**39.0 (38.5,39.4)**	**38.6 (38.2,38.8)**	**0.00038**	39.2 (38.5,39.7)	38.6 (38.1,38.8)	0.043
Oxygen saturation <93%	**21 (36.8%)**	**63 (32.5%)**	**0.53**	11 (32.4%)	52 (32.5%)	1.00
NA	**24**	**5**		1	4	
Tachypnea	**62 (87.3%)**	**123 (86.6%)**	**1.00**	15 (83.3%)	108 (87.1%)	0.71
NA	**10**	**57**		17	40	
Dyspnea	**22 (27.2%)**	**65 (32.8%)**	**0.39**	10 (28.6%)	55 (33.7%)	0.69
NA	**0**	**1**		0	1	
Cyanosis	**1 (1.2%)**	**1 (0.5%)**	**0.50**	0 (0.0%)	1 (0.6%)	1.00
NA	**0**	**2**		0	2	
Other signs at presentation						
Grunting	**0 (0.0%)**	**3 (1.5%)**	**0.56**	0 (0.0%)	3 (1.9%)	1.00

Variable	2015-2020 (pre-NPI) (*n* = 81)	2022-2025 (post-NPI) (*n* = 199)	p value	2023-2024 (post-NPI first-year peak) (*n* = 35)	2024-2025 (post-NPI second-year peak) (*n* = 164)	p value
Nasal flaring	**5 (6.2%)**	**8 (4.1%)**	**0.53**	1 (2.9%)	7 (4.3%)	1.00
Retractions	**25 (30.9%)**	**55 (27.9%)**	**0.66**	6 (17.1%)	49 (30.2%)	0.15
Abnormal auscultatory lung findings	**63 (77.8%)**	**159 (80.7%)**	**0.62**	26 (74.3%)	133 (82.1%)	0.34
Pharyngitis/Tonsillitis	**34 (42.0%)**	**86 (43.7%)**	**0.89**	18 (51.4%)	68 (42.0%)	0.35
Otitis media	**6 (7.4%)**	**30 (15.2%)**	**0.11**	5 (14.3%)	25 (15.4%)	1.00
Enlarged lymph nodes	**15 (18.5%)**	**27 (13.7%)**	**0.36**	5 (14.3%)	22 (13.6%)	1.00
Dermatological findings	**18 (22.2%)**	**18 (9.1%)**	**0.0053**	6 (17.1%)	12 (7.4%)	0.099
Dermatological prodrome (days)	**1.0 (0.0,2.0)**	**2.0 (0.0,5.0)**	**0.68**	5.0 (5.0,5.0)	1.5 (0.0,2.0)	0.31
NA	**7**	**11**		5	6	
Neurological findings	**2 (2.5%)**	**5 (2.5%)**	**1.00**	0 (0.0%)	5 (3.1%)	0.59
Neurological prodrome (days)	**1.0 (0.0,2.0)**	**0.5 (0.0,2.0)**	**1.00**	NA	0.5 (0.0,2.0)	NA
NA	**0**	**1**		0	1	
NA	**0**	**2**		0	2	
***Radiographic findings*[b]**						
Chest radiograph performed	**76 (93.8%)**	**138 (69.3%)**	**< 0.0001**	28 (80.0%)	110 (67.1%)	0.16
Pulmonary infiltrate in chest radiograph	**64 (85.3%)**	**120 (87.6%)**	**0.67**	24 (85.7%)	96 (88.1%)	0.75
Infiltrate type			**0.0041**			0.60
Alveolar (consolidation)	**52 (81.3%)**	**114 (95.0%)**		24 (100.0%)	90 (93.8%)	
Interstitial	**12 (18.8%)**	**6 (5.0%)**		0 (0.0%)	6 (6.3%)	
Pleural effusion	**22 (29.3%)**	**64 (46.7%)**	**0.019**	17 (60.7%)	47 (43.1%)	0.14
NA	**1**	**1**		0	1	
Diagnosis						
Pneumonia	**77 (95.1%)**	**172 (86.4%)**	**0.037**	28 (80.0%)	144 (87.8%)	0.27
Other LRTI	**2 (2.5%)**	**7 (3.5%)**	**1.00**	1 (2.9%)	6 (3.7%)	1.00
Not specified LRTI	**2 (2.5%)**	**20 (10.1%)**	**0.047**	6 (17.1%)	14 (8.5%)	0.13
Obstructive component	**8 (9.9%)**	**44 (22.1%)**	**0.018**	5 (14.3%)	39 (23.8%)	0.27
Extrapulmonary manifestation						
Total	**24 (29.6%)**	**36 (18.1%)**	**0.038**	10 (28.6%)	26 (15.9%)	0.091
Dermatological	**21 (25.9%)**	**31 (15.6%)**	**0.061**	8 (22.9%)	23 (14.0%)	0.20
Cutaneous involvement	**18 (85.7%)**	**20 (64.5%)**	**0.12**	4 (50.0%)	16 (69.6%)	0.41
Mucosal involvement	**8 (38.1%)**	**12 (38.7%)**	**1.00**	5 (62.5%)	7 (30.4%)	0.21
Neurological	**3 (3.7%)**	**2 (1.0%)**	**0.15**	0 (0.0%)	2 (1.2%)	1.00
Gastrointestinal	**1 (1.2%)**	**3 (1.5%)**	**1.00**	2 (5.7%)	1 (0.6%)	0.08
Other	**2 (2.5%)**	**0 (0.0%)**	**0.083**	0 (0.0%)	0 (0.0%)	1.00
Antibiotic treatment after presentation						
Total	**73 (90.1%)**	**168 (86.2%)**	**0.43**	29 (82.9%)	139 (86.9%)	0.59
Amoxicillin ± clavulanic acid	**37 (45.7%)**	**30 (15.4%)**	**< 0.0001**	3 (8.6%)	27 (16.9%)	0.30
Clarithromycin	**30 (37.0%)**	**92 (47.2%)**	**0.14**	10 (28.6%)	82 (51.3%)	0.016

Variable	2015-2020 (pre-NPI) (*n* = 81)	2022-2025 (post-NPI) (*n* = 199)	p value	2023-2024 (post-NPI first-year peak) (*n* = 35)	2024-2025 (post-NPI second-year peak) (*n* = 164)	p value
Azithromycin	**0 (0.0%)**	**3 (1.5%)**	**0.56**	1 (2.9%)	2 (1.3%)	0.45
Doxycycline	**24 (29.6%)**	**72 (36.9%)**	**0.27**	17 (48.6%)	55 (34.4%)	0.13
Other	**7 (8.6%)**	**8 (4.1%)**	**0.15**	4 (11.4%)	4 (2.5%)	0.036
NA	**0**	**4**		0	4	
Steroid treatment after presentation						
Total	**8 (10.3%)**	**51 (26.4%)**	**0.0032**	6 (18.2%)	45 (28.1%)	0.28
Systemic	**6 (7.7%)**	**43 (22.3%)**	**0.0048**	5 (15.2%)	38 (23.8%)	0.36
Inhaled	**0 (0.0%)**	**11 (5.7%)**	**0.037**	1 (3.0%)	10 (6.3%)	0.69
Other	**4 (5.1%)**	**5 (2.6%)**	**0.28**	1 (3.0%)	4 (2.5%)	1.00
NA	**3**	**6**		2	4	
Outcome						
Hospitalization	**35 (43.8%)**	**87 (43.7%)**	**1.00**	15 (42.9%)	72 (43.9%)	1.00
NA	**1**	**0**		0	0	
LOS (days)	**4.0 (3.0,5.0)**	**4.0 (2.0,5.0)**	**0.51**	3.0 (2.5,5.0)	4.0 (2.0,5.0)	0.86
NA	**1**	**4**		3	1	
ICU	**3 (3.8%)**	**11 (5.5%)**	**0.76**	2 (5.7%)	9 (5.5%)	1.00
NA	**1**	**0**		0	0	
Long-term sequelae[c]						
Total	**3 (4.5%)**	**4 (8.0%)**	**0.46**	1 (7.7%)	3 (8.1%)	NA
Death	**0 (0.0%)**	**0 (0.0%)**	**1.00**	0 (0.0%)	0 (0.0%)	1.00
Respiratory[d]	**2 (3.0%)**	**2 (4.0%)**	**1.00**	0 (0.0%)	2 (5.4%)	1.00
Dermatological[e]	**0 (0.0%)**	**2 (4.0%)**	**0.18**	1 (7.7%)	1 (2.7%)	0.46
Gastrointestinal[f]	**0 (0.0%)**	**2 (4.0%)**	**0.18**	0 (0.0%)	2 (5.4%)	1.00
Neurological	**0 (0.0%)**	**0 (0.0%)**	**1.00**	0 (0.0%)	0 (0.0%)	1.00
Other or not specified[g]	**1 (1.5%)**	**2 (4.0%)**	**0.58**	1 (7.7%)	1 (2.7%)	0.46
NA	**15**	**149**		22	127	0.086
**CO-DETECTION OF OTHER BACTERIA AND/OR VIRUSES IN THE RESPIRATORY TRACT**[h]						
Total	**14 (17.3%)**	**71 (35.7%)**	**0.0025**	11 (31.4%)	60 (36.6%)	0.70
Bacterial co-detection[i]						
Total	**2 (2.5%)**	**9 (4.5%)**	**0.52**	3 (8.6%)	6 (3.7%)	0.20
Chlamydia pneumoniae	**1 (1.2%)**	**3 (1.5%)**	**1.00**	2 (5.7%)	1 (0.6%)	0.08
Bordetella pertussis	**0 (0.0%)**	**1 (0.5%)**	**1.00**	0 (0.0%)	1 (0.6%)	1.00
Bordetella parapertussis	**0 (0.0%)**	**0 (0.0%)**	**1.00**	0 (0.0%)	0 (0.0%)	1.00
Other[j]	**1 (1.2%)**	**5 (2.5%)**	**0.68**	1 (2.9%)	4 (2.4%)	1.00
Viral co-detection						
Total	**13 (16.0%)**	**68 (34.2%)**	**0.0022**	9 (25.7%)	59 (36.0%)	0.33
Influenza virus	**0 (0.0%)**	**2 (1.0%)**	**1.00**	0 (0.0%)	2 (1.2%)	1.00
A	**0**	**1**	**1.00**	0	1	1.00
B	**0**	**0**	**1.00**	0	0	1.00
RSV	**2 (2.5%)**	**6 (3.0%)**	**1.00**	0 (0.0%)	6 (3.7%)	0.59
Coronavirus	**1 (1.2%)**	**12 (6.0%)**	**0.12**	2 (5.7%)	10 (6.1%)	1.00

Variable	2015-2020 (pre-NPI) (*n* = 81)	2022-2025 (post-NPI) (*n* = 199)	p value	2023-2024 (post-NPI first-year peak) (*n* = 35)	2024-2025 (post-NPI second-year peak) (*n* = 164)	p value
229E	**0**	**0**	**1.00**	0	0	1.00
HKU1	**0**	**1**	**1.00**	1	0	0.18
NL63	**0**	**3**	**0.56**	0	3	1.00
OC43	**1**	**1**	**0.50**	0	1	1.00
MERS	**0**	**0**	**1.00**	0	0	1.00
SARS	**0**	**8**	**0.11**	1	7	1.00
Adenovirus	**6 (7.4%)**	**5 (2.5%)**	**0.084**	0 (0.0%)	5 (3.0%)	0.59
Human Metapneumovirus	**0 (0.0%)**	**0 (0.0%)**	**1.00**	0 (0.0%)	0 (0.0%)	1.00
Human Rhinovirus/Enterovirus	**5 (6.2%)**	**52 (26.1%)**	**< 0.0001**	7 (20.0%)	45 (27.4%)	0.41
Parainfluenza virus	**0 (0.0%)**	**11 (5.5%)**	**0.037**	2 (5.7%)	9 (5.5%)	1.00
1	**0**	**0**	**1.00**	0	0	1.00
2	**0**	**6**	**0.19**	0	6	0.59
3	**0**	**4**	**0.33**	2	2	0.14
4	**0**	**2**	**1.00**	0	2	1.00
Bocavirus	**2 (2.5%)**	**0 (0.0%)**	**0.083**	0 (0.0%)	0 (0.0%)	1.00

The annual figures always refer to the 12-month period April 1 to March 31 (e.g., April 1, 2015–March 31, 2016). Continuous variables are summarized as median (1st quartile, 3rd quartile), categorical variables as no. (%) or no. P values were calculated by the Kruskal–Wallis rank sum test (continuous variables) or Fisher's exact test (categorical variables). **Abbreviations:** ICU, intensive-care unit LOS, length of stay; LRTI, lower respiratory tract infection; NA, not available; NPI, non-pharmaceutical intervention; PCR, polymerase chain reaction; RTI, respiratory tract infection; URTI, upper respiratory tract infection.

[a] Symptoms within ± 30 days of the patient's symptom onset.

[b] Chest radiographs originating from an earlier presentation were excluded.

[c] Pre-NPI, one patient was later diagnosed with juvenile idiopathic arthritis, and post-NPI one patient was later diagnosed with postural orthostatic tachycardia syndrome (POTS) and gastritis. These diagnoses were not considered long-term sequelae.

[d] Pre-NPI: bronchiolitis obliterans (*n*=1), unspecific exertional dyspnea (*n*=1); post-NPI: unspecific cough (*n*=1), unspecific exertional dyspnea/cough/chest pain (*n*=1).

[e] Postinflammatory pigmentary alteration (*n*=1), mucosal inflammation (*n*=1).

[f] Colitis with stenosis (*n*=1), unspecific abdominal pain/nausea (*n*=1).

[g] Pre-NPI: not specified (*n*=1), post-NPI: reduced physical capacity (*n*=1), fatigue (*n*=1).

[h] *M. pneumoniae*-specific testing with singleplex PCR has been replaced by multiplex PCR as of October 12, 2020 (supplementary appendix 3 in [1], page 16).

[i] For patients pre-NPI who had participated in the myCAP study, singleplex *Streptococcus pneumoniae*-specific PCR results performed as part of that study were excluded from the analyses to avoid testing bias.

[j] Pre-NPI: *Staphylococcus aureus* / *Haemophilus influenzae* (*n*=1); post-NPI: *Streptococcus pneumoniae* (*n*=1), *Haemophilus parainfluenzae* / *Rothia mucilaginosa* / *α-hemolytic streptococci* (*n*=1), *Streptococcus pneumoniae* / *Haemophilus influenzae* / *Acinteobacter junii* (*n*=1), *Haemophilus influenzae* (*n*=1), *Staphylococcus aureus* (*n*=1).

Supplementary Table S11 Adjusted binomial generalized linear model for hospitalization with logit link and cohort (pre- versus post-NPI) as explanatory variable of children with *Mycoplasma pneumoniae* detection by PCR from April 1, 2015 to March 31, 2025

Characteristic	OR	95% CI	p-value
2022-2025 (post-NPI)	0.72	0.42, 1.23	0.22
Age (years)	0.90	0.84, 0.95	0.00062
Female sex	1.21	0.75, 1.94	0.43
Underlying diseases	2.56	1.48, 4.47	0.00082

The annual figures always refer to the 12-month period April 1 to March 31 (e.g., April 1, 2015–March 31, 2016). **Abbreviations**: CI, confidence interval; NPI, non-pharmaceutical intervention; OR, odds ratio.

Supplementary Table S12 Unadjusted binomial generalized linear model for hospitalization with logit link and cohort (pre- versus post-NPI) as explanatory variable of children with *Mycoplasma pneumoniae* detection by PCR from April 1, 2015 to March 31, 2025

Characteristic	OR	95% CI	p-value
2022-2025 (post-NPI)	0.80	0.48, 1.34	0.40

The annual figures always refer to the 12-month period April 1 to March 31 (e.g., April 1, 2015–March 31, 2016). **Abbreviations**: CI, confidence interval; NPI, non-pharmaceutical intervention; OR, odds ratio.

Supplementary Table S13 Adjusted binomial generalized linear model for hospitalization with logit link and cohort (first- versus second-year peak post-NPI) as explanatory variable of children with *Mycoplasma pneumoniae* detection by PCR from April 1, 2023 to March 31, 2025

Characteristic	OR	95% CI	p-value
2024-2025 (post-NPI second-year peak)	0.50	0.23, 1.09	0.082
Age (years)	0.86	0.79, 0.92	< 0.0001
Female sex	1.53	0.87, 2.70	0.14
Underlying diseases	2.49	1.35, 4.66	0.0038

The annual figures always refer to the 12-month period April 1 to March 31 (e.g., April 1, 2015–March 31, 2016). **Abbreviations**: CI, confidence interval; NPI, non-pharmaceutical intervention; OR, odds ratio.

Supplementary Table S14 Unadjusted binomial generalized linear model for hospitalization with logit link and cohort (first- versus second-year peak post-NPI) as explanatory variable of children with *Mycoplasma pneumoniae* detection by PCR from April 1, 2023 to March 31, 2025

Characteristic	OR	95% CI	p-value
2024-2025 (post-NPI second-year peak)	0.70	0.34, 1.43	0.32

The annual figures always refer to the 12-month period April 1 to March 31 (e.g., April 1, 2015–March 31, 2016). **Abbreviations**: CI, confidence interval; NPI, non-pharmaceutical intervention; OR, odds ratio.

Supplementary Table S15 Adjusted binomial generalized linear model for ICU admission with logit link and cohort (pre- versus post-NPI) as explanatory variable of children with *Mycoplasma pneumoniae* detection by PCR from April 1, 2015 to March 31, 2025

Characteristic	OR	95% CI	p-value
2022-2025 (post-NPI)	0.90	0.29, 3.34	0.86
Age (years)	0.95	0.83, 1.09	0.48
Female sex	1.12	0.39, 3.15	0.83
Underlying diseases	2.83	0.96, 8.09	0.051

The annual figures always refer to the 12-month period April 1 to March 31 (e.g., April 1, 2015–March 31, 2016). **Abbreviations**: CI, confidence interval; NPI, non-pharmaceutical intervention; OR, odds ratio.

Supplementary Table S16 Unadjusted binomial generalized linear model for ICU admission with logit link and cohort (pre- versus post-NPI) as explanatory variable of children with *Mycoplasma pneumoniae* detection by PCR from April 1, 2015 to March 31, 2025

Characteristic	OR	95% CI	p-value
2022-2025 (post-NPI)	1.04	0.35, 3.82	0.94

The annual figures always refer to the 12-month period April 1 to March 31 (e.g., April 1, 2015–March 31, 2016). **Abbreviations**: CI, confidence interval; NPI, non-pharmaceutical intervention; OR, odds ratio.

Supplementary Table S17 Adjusted binomial generalized linear model for ICU admission with logit link and cohort (first- versus second-year peak post-NPI) as explanatory variable of children with *Mycoplasma pneumoniae* detection by PCR from April 1, 2023 to March 31, 2025

Characteristic	OR	95% CI	p-value
2024-2025 (post-NPI second-year peak)	0.48	0.13, 2.30	0.30
Age (years)	0.92	0.78, 1.07	0.28
Female sex	1.02	0.29, 3.45	0.97
Underlying diseases	2.97	0.87, 10.2	0.076

The annual figures always refer to the 12-month period April 1 to March 31 (e.g., April 1, 2015–March 31, 2016). **Abbreviations**: CI, confidence interval; NPI, non-pharmaceutical intervention; OR, odds ratio.

Supplementary Table S18 Unadjusted binomial generalized linear model for ICU admission with logit link and cohort (first- versus second-year peak post-NPI) as explanatory variable of children with *Mycoplasma pneumoniae* detection by PCR from April 1, 2023 to March 31, 2025

Characteristic	OR	95% CI	p-value
2024-2025 (post-NPI second-year peak)	0.54	0.15, 2.51	0.37

The annual figures always refer to the 12-month period April 1 to March 31 (e.g., April 1, 2015–March 31, 2016). **Abbreviations**: CI, confidence interval; NPI, non-pharmaceutical intervention; OR, odds ratio.
